# Cardiac magnetic resonance and computed tomography angiography for clinical imaging of stable coronary artery disease. Diagnostic classification and risk stratification

**DOI:** 10.3389/fphys.2014.00291

**Published:** 2014-08-06

**Authors:** Grigorios Korosoglou, Sorin Giusca, Gitsios Gitsioudis, Christian Erbel, Hugo A. Katus

**Affiliations:** Department of Cardiology, University of HeidelbergHeidelberg, Germany

**Keywords:** coronary artery disease, atherosclerotic plaque, coronary computed tomography, cardiac magnetic resonance, risk stratification

## Abstract

Despite advances in the pharmacologic and interventional treatment of coronary artery disease (CAD), atherosclerosis remains the leading cause of death in Western societies. X-ray coronary angiography has been the modality of choice for diagnosing the presence and extent of CAD. However, this technique is invasive and provides limited information on the composition of atherosclerotic plaque. Coronary computed tomography angiography (CCTA) and cardiac magnetic resonance (CMR) have emerged as promising non-invasive techniques for the clinical imaging of CAD. Hereby, CCTA allows for visualization of coronary calcification, lumen narrowing and atherosclerotic plaque composition. In this regard, data from the CONFIRM Registry recently demonstrated that both atherosclerotic plaque burden and lumen narrowing exhibit incremental value for the prediction of future cardiac events. However, due to technical limitations with CCTA, resulting in false positive or negative results in the presence of severe calcification or motion artifacts, this technique cannot entirely replace invasive angiography at the present time. CMR on the other hand, provides accurate assessment of the myocardial function due to its high spatial and temporal resolution and intrinsic blood-to-tissue contrast. Hereby, regional wall motion and perfusion abnormalities, during dobutamine or vasodilator stress, precede the development of ST-segment depression and anginal symptoms enabling the detection of functionally significant CAD. While CT generally offers better spatial resolution, the versatility of CMR can provide information on myocardial function, perfusion, and viability, all without ionizing radiation for the patients. Technical developments with these 2 non-invasive imaging tools and their current implementation in the clinical imaging of CAD will be presented and discussed herein.

## Introduction

**Figure 1:**
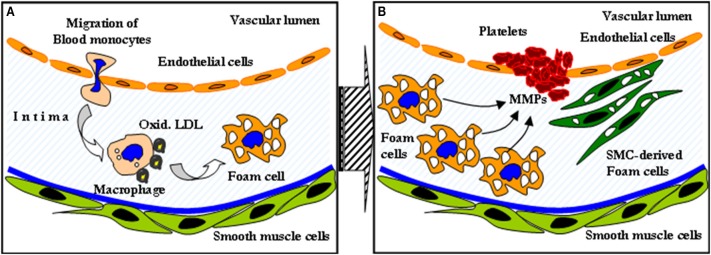
**The role of inflammation in atherosclerotic disease**. Macrophages engulfing oxidized LDL transform to foam cells **(A)**. The secretion of metalloptroteinases subsequently causes destabilization of cap tissues, resulting in plaque rupture **(B)**.

Despite major advances in the treatment of coronary artery disease (CAD), it still remains one of the leading causes of death in Western societies (Murray and Lopez, 1997; Myerburg et al., 1997; Naghavi et al., 2003). The role of inflammation and the key role of plaque macrophages in all stages of atherosclerosis is widely appreciated (Libby et al., 2002; Swirski et al., 2006). Such inflamed atherosclerotic plaques typically exhibit a lipid-rich pool, overexpression of collagenases and metalloptroteinases and a thin fibrotic cap (van Rugge et al., 1993). The secretion of proteolytic enzymes by lesional macrophages can cause destabilization of cap tissues, resulting in plaque rupture (Figure 1).

X-ray coronary angiography is the current clinical gold-standard technique for the detection of CAD. Coronary angiography provides an accurate measure of stenosis. However, this technique is invasive and almost entirely relies on anatomic structure of the vascular lumen (Vallabhajosula and Fuster, 1997), providing limited information on the composition of the arterial wall (Fuster et al., 1992; Topol and Nissen, 1995). Furthermore, the phenomenon of vascular remodeling decreases the sensitivity of coronary angiography to detect the true atherosclerotic plaque burden. Revascularization techniques of coronary artery bypass surgery (CABG) and percutaneous coronary interventions (PCI) are currently guided by findings provided by X-ray angiography, and therefore target high-grade coronary lesions. However, acute coronary syndromes most commonly result from the rupture of plaques that are angiographically modest in severity (*non-critical stenoses*) (Fuster et al., 1992; Korosoglou et al., 2008a). Therefore, the currently used revascularization strategies may do little to prevent future coronary events (Libby, 2001; Boden et al., 2007).

## Cardiac magnetic resonance imaging

*Cardiac Magnetic Resonance (CMR)* can provide non-invasive imaging with sub-millimeter spatial resolution and high blood-to-tissue contrast (Toussaint et al., 1996). Due to its non-invasive nature, high intrinsic contrast and the absence of radiation exposure for the patients, CMR is to date the preferred technique for the assessment of cardiac morphology and function. In this regard, the versatility of CMR can provide assessment of myocardial function, perfusion, viability and if required cardiac metabolism within a single “one stop shop” non-invasive examination. Furthermore, its tomographic nature provides excellent comparability between perfusion and function of corresponding myocardial segments and high reproducibility of the acquired results.

### Diagnosis of coronary artery disease

Modern CMR 1.5 & 3.0 Tesla clinical scanners provide high temporal (~40ms) and spatial (<1.0^*^1.0 mm in plane) resolution for the assessment of cardiac function. The latter is almost 10-fold higher compared to competing nuclear scintigraphy techniques. Although earlier CMR studies had limitations, such as poor slice coverage and low temporal resolution, limiting the detection of CAD, subsequent data demonstrated that CMR compares favorably to SPECT for the detection of myocardial ischemia (Schwitter et al., 2001, 2012, 2013; Wagner et al., 2003). In a recent prospective, real-world clinical trial, which included 752 consecutive patients, CMR exhibited significantly higher diagnostic accuracy compared to SPECT for CAD detection (Greenwood et al., 2012).

**Figure 2:**
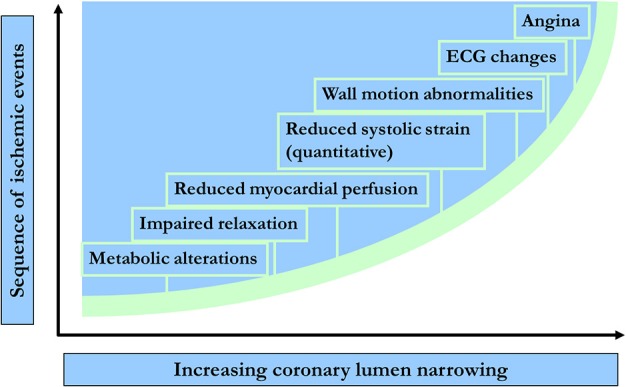
**Temporal sequence of events in terms of metabolic & myocardial dysfunction during ischemia**.

**Figure 3:**
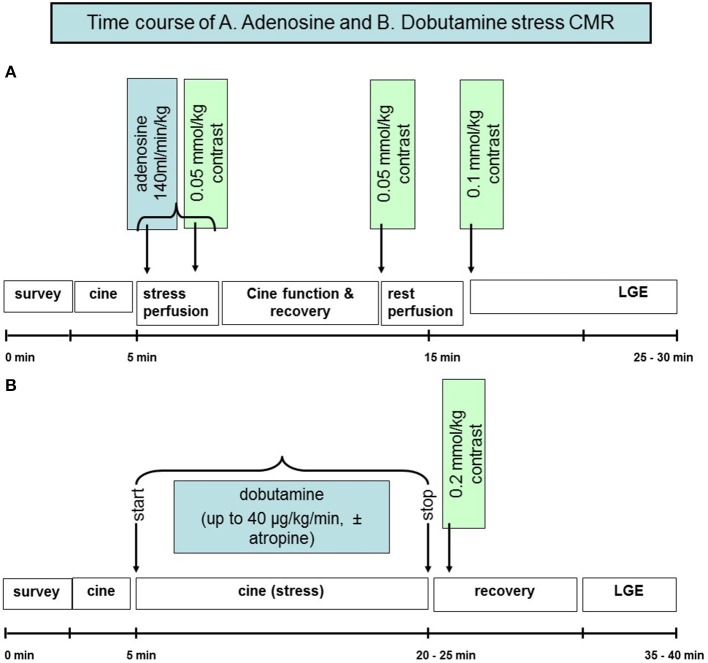
**Flow chart illustrating the time course of adenosine perfusion and dobutamine stress CMR examinations (A,B)**.

Detection of CAD using CMR is mainly based on the evaluation of the functional significance of coronary artery stenosis during pharmacologic stress testing with either adenosine or dipyridamole, i.e., coronary vasodilators, or dobutamine, which is a synthetic ß-adrenergic stimulator. Using stress CMR, inducible myocardial ischemia can be detected in form of inducible wall motion abnormalities during dobutamine stress or in form of perfusion deficits during vasodilator stress, which both precede the development of ST-segment depression and anginal symptoms in the ischemic cascade (Figure 2) and have been reported to accurately detect functionally significant CAD (Pennell et al., 1990, 1992; van Rugge et al., 1993, 1994; Hundley et al., 1999; Nagel et al., 1999, 2003; Schwitter et al., 2001; Schalla et al., 2002; Doyle et al., 2003; Ishida et al., 2003; Rerkpattanapipat et al., 2003; Giang et al., 2004; Kawase et al., 2004; Paetsch et al., 2004, 2006; Plein et al., 2004, 2005; Takase et al., 2004; Sakuma et al., 2005; Cury et al., 2006; Jahnke et al., 2006; Klem et al., 2006; Pilz et al., 2006; reviewed in Nandalur et al., 2007). Flow charts for these 2 forms of pharmacologic stress CMR with corresponding time spent are illustrated in Figures 3A,B for dobutamine and adenosine stress CMR, respectively. Due to its tomographic nature, CMR allows for sequential acquisition of identical slices (each segment at baseline serves as its own control during peak stress), providing the reproducible and accurate assessment of wall motion and strain response during dobutamine stress testing. Similarly, myocardial perfusion can be assessed in identical slices during baseline and pharmacologic hyperemia.

In a meta-analysis including 14 dobutamine and 24 adenosine stress CMR studies with 754 and 1516 patients, respectively sensitivities and specificities of 83% (95% confidence interval of 79–88%), 86% (95% confidence interval of 81–91%) and of 91% (95% confidence interval of 88–94%) and 81% (95% confidence interval of 77–85%), respectively were reported (Tables 1, 2). Especially after exclusion of studies utilizing dipyridamole as a stressor, the assessment of inducible wall motion abnormalities exhibited an improved sensitivity of 85% (95% confidence interval of 82–90%) without a loss in terms of specificity. Furthermore, in a direct comparison between adenosine and dobutamine stress CMR in 79 consecutive patients with suspected or known CAD, the analysis of inducible wall motion abnormalities during dobutamine CMR offered the best trade-off between sensitivity and specificity (Paetsch et al., 2004). In this regard, the latter provided sensitivity of 89% and specificity of 80% for the identification of patients with significant CAD.

**Table 1:** **Studies performed with dobutamine stress CMR for the detection of CAD by inducible wall motion abnormalities**.

**Study**	**Stressor(s)**	**Number of patients**	**MR-scanner**	**Definition of relevant stenosis (%)**	**Sensitivity (%) with 95%CI**	**Specificity (%) with 95%CI**
Hundley et al., 1999	Dobutamine/Atropin	41	GE 1.5T	>50	83 (86–93)	83 (36–100)
Jahnke et al., 2006	Dobutamine	40	Philips 1.5T	≥50	83 (51–97)	89 (71–97)
Nagel et al., 1999	Dobutamine	172	Philips 1.5T	≥50	86 (78–92)	86 (75–93)
Paetsch et al., 2004	Dobutamine/Atropin	79	Philips 1.5T	>50	89 (77–96)	81 (61–93)
Paetsch et al., 2006	Dobutamine	150	Philips 1.5T	≥50	78 (67–87)	88 (78–94)
Pennell et al., 1992	Dobutamine	25	Picker 0.5T	≥50	91 (71–99)	100 (29–100)
Rerkpattanapipat et al., 2003	Exercise	27	GE 1.5T	>70	79 (49–95)	85 (55–98)
Schalla et al., 2002	Dobutamine	22	Philips 1.5T	>75	81 (54–96)	83 (36–100)
van Rugge et al., 1993	Dobutamine	45	Philips 1.5T	>50	81 (65–92)	100 (63–100)
van Rugge et al., 1994	Dobutamine	39	Philips 1.5T	≥50	91 (76–98)	83 (36–100)
Pooled data	Dobutamine ± Atropin	680		≥50–75	85 (82–90)	86 (81–91)

**Table 2:** **Vasodilator stress perfusion CMR for the detection of CAD**.

**Study**	**Stressor(s)**	**Number of patients**	**MR-scanner**	**Definition of relevant stenosis (%)**	**Sensitivity (%) with 95%CI**	**Specificity (%) with 95%CI**
Cury et al., 2006	Dipyridamole	47	GE 1.5T	≥70	87 (74–94)	89 (80–95)
Doyle et al., 2003	Dipyridamole	199	Philips 1.5T	≥70	58 (37–77)	78 (71–84)
Giang et al., 2004	Adenosine	44	GE 1.5T	≥50	93 (77–99)	75 (48–92)
Pennell et al., 1990	Dipyridamole	40	Picker 0.5T	Not specified	62 (45–77)	100 (3–100)
Ishida et al., 2003	Dipyridamole	104	GE 1.5T	≥70	90 (81–95)	85 (67–94)
Kawase et al., 2004	Nicorandil	50	Philips 1.5T	>70	94 (80–99)	94 (71–100)
Klem et al., 2006	Adenosine	95	Siemens 1.5T	≥70	89 (75–97)	87 (76–95)
Nagel et al., 2003	Adenosine	90	Philips 1.5T	≥75	88 (75–96)	90 (77–97)
Pilz et al., 2006	Adenosine	176	GE 1.5T	>70	96 (91–99)	83 (71–91)
Plein et al., 2004	Adenosine	71	Philips 1.5T	≥70	96 (88–100)	83 (52–98)
Plein et al., 2005	Adenosine	92	Philips 1.5T	>70	88 (77–95)	82 (52–90)
Sakuma et al., 2005	Dipyridamole	40	Siemens 1.5T	>70	81 (58–95)	68 (43–87)
Schwitter et al., 2001	Dipyridamole	48	GE 1.5T	≥50	87 (71–95)	85 (35–93)
Takase et al., 2004	Dipyridamole	102	GE 1.5T	>50	93 (85–98)	85 (65–96)
Paetsch et al., 2004	Adenosine	79	Philips 1.5T	>50	91 (79–97)	62 (41–80)
Pooled data	Vasodilator stress	1237			91 (88–94)	81 (77–85)

**Figure 4:**
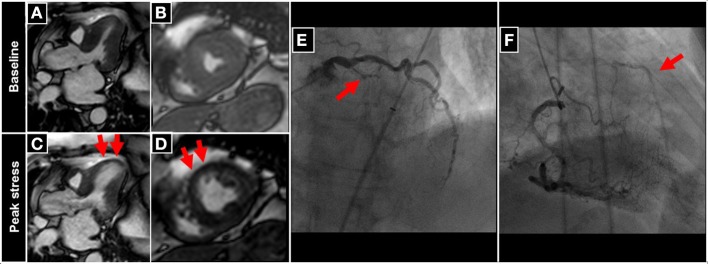
**Patient undergoing DCMR for suspected progression of CAD after PCI and stent placement in the LAD two years ago**. During peak DCMR inducible wall motion abnormality of the LV-apex and anterior-septal wall can be observed (normal wall motion at baseline in **A,B** and inducible akinesia during peak stress (red arrows in **C,D**). Subsequent coronary angiography demonstrates collateralized occlusion of the LAD (red arrows in **E,F**).

An example of a patient with inducible wall motion abnormality of the anterior-septal apical wall during peak dobutamine stress CMR is illustrated in Figures 4A–D. Corresponding angiographic images, demonstrating collateralized occlusion of the LAD can be appreciated in Figures 4E,F.

Despite the high diagnostic value and reproducibility of stress CMR (Paetsch et al., 2006), and its in the meanwhile wide acceptance among cardiologists in the clinical practice, the assessment of cine images relies on visual interpretation of regional wall motion, which is subjective and depends on the experience of the readers. Recently, Strain-Encoded-MRI (SENC) has been proposed for the objective color coded evaluation of regional myocardial strain. Using SENC, myocardial deformation can be assessed visually using a color coded scale, whereas strain and strain rate can be quantified in circumferential and longitudinal direction. The ability of SENC to quantify myocardial strain has been validated in experimental and in clinical settings (Kraitchman et al., 2003; Pan et al., 2006; Korosoglou et al., 2008b, 2009a,b,c, 2010a, 2011a; Youssef et al., 2008; Neizel et al., 2009) and this technique favorably compares over more conventional CMR tagging sequences in terms of temporal resolution, total scan duration and time-spent required for the post-processing of the acquired data. In a direct comparison of conventional tagging to SENC, the latter demonstrated non-inferior overall accuracy for the detection of functional significant CAD. Furthermore, studying 101 consecutive patients, we previously demonstrated the ability of the direct color coded visualization of strain on SENC images to enhance the sensitivity of dobutamine stress CMR (86 vs. 98%, *p* < 0.05) for the detection of obstructive CAD (Korosoglou et al., 2009b). Interestingly, quantitative myocardial strain rate already decreased with moderate 40–60% coronary artery stenosis, before relevant reduction of myocardial strain and the occurrence of evident inducible wall abnormalities by conventional cine imaging. In addition, SENC enabled the detection of functional significant CAD already during intermediate dobutamine stress CMR (at 20 μg/kg of body weight), which may provide improved patient safety within a lower time spent (Korosoglou et al., 2010a).

**Figure 5:**
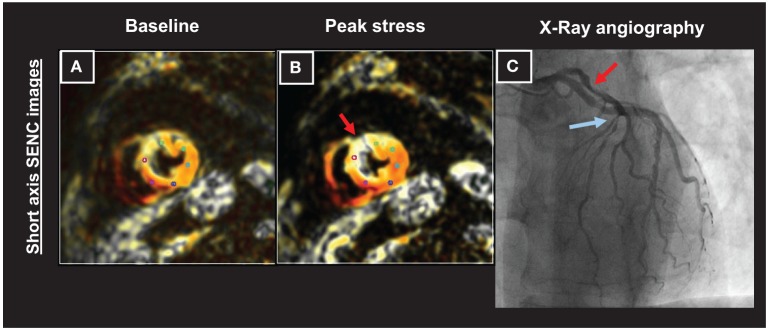
**Patient with an increasing strain abnormality in the anterior-septal wall during dobutamine stress CMR (A,B; red arrows in B)**. A high grade left anterior descending (LAD) stenosis was observed by coronary angiography (red and blue arrows depicting high grade lesions in the proximal and mid part of the artery, respectively in **C**).

An example of a patient with an increasing strain abnormality in the anterior-septal wall during dobutamine stress (Figures 5A,B; red arrows in Figure 5B) can be appreciated in Figure 5. The presence of a high grade left anterior descending (LAD) stenosis was confirmed by coronary angiography (red and blue arrows depicting high grade lesions in the proximal and mid part of the LAD, respectively in Figure 5C).

Similar to the assessment of wall motion abnormalities, most CMR centers perform visual analysis of perfusion scans for the diagnosis of inducible myocardial ischemia in the clinical routine. In this regard, the transmural extent of a perfusion deficit is determined from dynamic images showing the maximum extent of regional hypoenhancement. Hereby, increase in regional hypoenhancement during adenosine or dypiridamole stress (≥25% increase in hypoenhancement transmurality compared to baseline scans) in at least one myocardial segment, which persists for ≥5 consecutive image frames, is considered as indicative of inducible ischemia (Korosoglou et al., 2010b). Several studies also demonstrated the value of myocardial perfusion assessment during high dose dobutamine stress CMR for the detection of CAD, particularly in patients with left ventricular hypertrophy and concentric remodeling (Gebker et al., 2008, 2010). Recently, fully quantitative stress perfusion CMR was shown to exhibit higher precision for the detection of obstructive CAD (≥70% stenosis) compared to semi-quantitative and visual assessment (Mordini et al., 2014). Multi-center studies are now warranted, in order to investigate the potential of such fully quantitative stress perfusion algorithms for the diagnostic work-up of patients with suspected or known CAD in the clinical routine.

Recent studies have also demonstrated the cost-effectiveness of CMR in the clinical routine. Thus, an economic evaluation of the CE-MARC study, which showed that CMR has superior diagnostic accuracy to SPECT (Greenwood et al., 2012, 2014), demonstrated that CMR is also a cost-effective strategy in patients with suspected CAD (Walker et al., 2013). In addition, stress CMR was shown to represent an effective and cost-reducing strategy in the diagnostic work-up of patients with acute chest pain and intermediate likelihood for evolving acute coronary syndromes (Miller et al., 2011; Hall et al., 2012). Currently, the early implementation of CCTA and CMR in the diagnostic process of patients with suspected non-ST-elevation myocardial infarction and its influence in terms of diagnostic classification, outcomes, and cost-effectiveness is investigated in the CARMENTA trial (Smulders et al., 2013).

### Assessment of clinical outcomes

Estimating the risk for subsequent cardiac events is of paramount importance in patients with known or suspected CAD, because an invasive therapy is warranted for patients with myocardial ischemia who are at high-risk for future events according to current guidelines (Montalescot et al., 2013). Several studies have previously demonstrated the usefulness of both vasodilator perfusion and dobutamine stress CMR for the estimation of clinical outcomes in patients with CAD (Ingkanisorn et al., 2006; Bodi et al., 2007; Jahnke et al., 2007, 2012; Pilz et al., 2008; Doesch et al., 2009; Lerakis et al., 2009; Steel et al., 2009; Vogel-Claussen et al., 2009; Charoenpanichkit et al., 2010; Korosoglou et al., 2010b; Bingham and Hachamovitch, 2011; Coelho-Filho et al., 2011; Gebker et al., 2011, 2012; Kelle et al., 2011; Krittayaphong et al., 2011; Lo et al., 2011; Lubbers et al., 2012; Bertaso et al., 2013; Buckert et al., 2013, reviewed in Lipinski et al., 2013). A recent meta-analysis systematically analyzed these data, including 14 vasodilator, 4 dobutamine studies and 1 study using both adenosine and dobutamine in a total of over 11,500 patients and with a mean follow-up duration of over 2.5 years (Lipinski et al., 2013). In this large meta-analysis, patients with inducible ischemia by stress CMR exhibited a markedly higher risk for future cardiac events, including myocardial infarction (odds ratio = 7.7) and cardiovascular death (odds ratio = 7.0) compared to those without inducible wall motion or perfusion abnormalities during CMR stress testing. The number of patients, mean follow-up duration, proportion of patients with known CAD and positive stress test results, as well as the odds ratio for future cardiac events in patients with inducible wall motion abnormalities or perfusion defects are illustrated for each of these studies in Table 3.

**Table 3:** **Studies demonstrating the ability of dobutamine and vasodilator stress CMR for the assessment of clinical outcomes**.

**Study**	**Mean follow-up (years)**	**Number of patients**	**Known CAD (%)**	**Proportion of patients with positive stress (%)**	**Odds ratio in patients with positive CMR**
**DOBUTAMINE STRESS CMR**					
Charoenpanichkit et al., 2010	6.0	353	Not reported	31	3.1
Gebker et al., 2011	2.1	1167	48	40	11.3
Jahnke et al., 2007	2.3	513	54	41	4.7
Kelle et al., 2011	3.7	1463	52	30	2.9
Korosoglou et al., 2010b	2.0	1473	55	20	5.9
**VASODILATOR STRESS CMR**					
Bertaso et al., 2013	1.8	362	43	25	4.7
Bingham and Hachamovitch, 2011	2.6	908	49	33	1.76
Bodi et al., 2007	1.1	1722	Not reported	41	1.15
Buckert et al., 2013	4.2	1152	Not reported	27	3.21
Coelho-Filho et al., 2011	2.5	405	Not reported	31	17.2
Doesch et al., 2009	2.5	81	100	56	16.6
Ingkanisorn et al., 2006	1.3	135	17	21	30.0
Jahnke et al., 2012	4.8	679	54	48	4.1
Krittayaphong et al., 2011	2.9	1232	12	34	9.7
Lerakis et al., 2009	0.8	103	13	10	Non-estimable
Lo et al., 2011	3.2	203	16	21	7.7
Lubbers et al., 2012	1.8	125	Not reported	10	Non-estimable
Pilz et al., 2008	1.0	218	0	0	Non-estimable
Steel et al., 2009	1.4	254	Not reported	29	8.04
Vogel-Claussen et al., 2009	1.2	27	19	19	Non-estimable

The assessment of myocardial viability using late gadolinium enhancement (LGE) is an established method for the assessment of infarct size and for the risk stratification of patients after acute myocardial infarction and with chronic ischemic heart disease (Gerber et al., 2002; Selvanayagam et al., 2004; Giannitsis et al., 2008; Larose et al., 2010). Thus, the presence and extent of LGE in terms of infarct size and transmurality is predictive of functional recovery in patients after acute infarction as well as in patients with chronic infarction who undergo coronary revascularization procedures. More recently, LGE was shown to be a predictor of poor cardiac outcomes in patients with suspected CAD and without history of myocardial infarction. In such patients, even small amounts of subendocardial LGE carried a high risk for future infarction and cardiac death (Kwong et al., 2006). Furthermore, LGE has revealed a higher prevalence of such unrecognized infarction even in community-based cohorts of older individuals and is associated with increased mortality risk (Barbier et al., 2011; Schelbert et al., 2012), even if some unrecognized infarctions are very small. In addition, we and others recently demonstrated that LGE has complementary value to both adenosine perfusion and high dose dobutamine CMR for the assessment of CAD and clinical outcomes (Steel et al., 2009; Bingham and Hachamovitch, 2011; Kelle et al., 2013).

**Figure 6:**
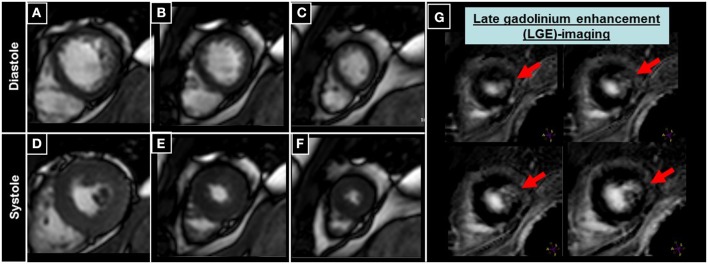
**Patient undergoing CMR with late gadolinium enhancement (LGE) imaging, where a small scar of the apical anterior-lateral wall can be appreciated (red arrows in **G**), without detectable wall motion abnormalities in the corresponding cine images **(A–F)****.

An example of a patient with a small unrecognized scar of the apical anterior-lateral wall without detectable wall motion abnormality by cine images can be appreciated in Figure 6.

Recent studies also demonstrated the ability of quantitative myocardial deformation assessment using SENC to estimate cardiac outcomes (Korosoglou et al., 2011a). Thus, patients with impaired myocardial deformation exhibited a higher rate of cardiac death and non-fatal myocardial infarction compared to those with preserved myocardial deformation by strain and strain rate (Korosoglou et al., 2011a). Hereby, strain rate seems to better reflect changes in regional myocardial contractile behavior, in contrast to alterations in systolic strain, which can be related to increased heart rate during dobutamine stress or to altered myocardial loading conditions (Weidemann et al., 2002).

Ongoing clinical trials such as the PROMISE study now aim at comparing functional stress tests such as CMR and echocardiography with anatomical modalities such as CCTA in order to determine which might be better at finding out who has heart disease and will require more testing and treatment (https://www.promisetrial.org/). More than 10,000 have already been enrolled in this study and first results are expected in 2015.

## Cardiac computed tomography (CTA)

*Cardiac Computed Tomography Angiography (CCTA)* can provide non-invasive imaging of moving coronary arteries with sub-millimeter spatial resolution and high signal-to-noise ratio. This technique has seen rapid progress in its utility for the assessment of CAD in the last years and can provide delineation of both vessel lumen and coronary vessel wall. The latter enables the assessment of atherosclerotic plaque composition, which seems to be of great importance not only for the diagnostic classification but also for the estimation of future cardiac events in patients with suspected CAD. In contrast to CMR, the administration of iodine contrast agents is necessary with CCTA for the visualization of cardiac structures and coronary arteries.

In addition CCTA is associated with radiation exposure for the patients, which limits its serial applicability, particularly in younger patients (Einstein et al., 2007). This still raises concerns among physicians and radiologists with CCTA, as it may be associated with non-negligible lifetime attributable risk of breast or lung cancer. Therefore, several strategies have been developed lately in order to reduce the resultant radiation dosages (Hausleiter et al., 2010; Hosch et al., 2011, 2012).

### Diagnosis of coronary artery disease

Several studies have demonstrated the ability of CCTA to detect anatomically significant CAD with high negative predictive value and clinically acceptable overall accuracy. Previous studies performed with 16-slice CT scanners, already pointed to the high negative predictive value of the technique (Nieman et al., 2002; Ropers et al., 2003; Kuettner et al., 2004; Mollet et al., 2004; Dewey et al., 2006), i.e., its ability to exclude relevant CAD with high accuracy (Table 4A). Subsequent studies, performed with 64-slice scanners confirmed the high negative predictive value of CCTA for the exclusion of anatomically relevant CAD, showing overall increased accuracy compared to previous studies (Heuschmid et al., 2007; Leber et al., 2007; Meijboom et al., 2007; Ropers et al., 2007; Shabestari et al., 2007; Miller et al., 2008), (Table 4B). Importantly, the ability of CCTA to detect CAD using X-Ray angiography as a gold standard is related to the pre-test probability of the study cohort. In this regard, a negative CCTA scan can reliably rule out the presence of significant CAD in patients with a low or intermediate likelihood for CAD, whereas it does not necessarily provide additional diagnostic information in patients with high pre-test probability (Meijboom et al., 2007). This reduction of the negative predictive values in patients with high disease prevalence was also shown in a comparable study comprising 88 patients (Husmann et al., 2008). Such high-risk symptomatic patients are likely to proceed to invasive angiography even if CCTA is negative, since the post-test probability of significant CAD is still >10%. CCTA therefore appears to have limited applicability in such patients, who should rather undergo a functional test as stress CMR for the assessment of inducible myocardial ischemia prior to invasive diagnostics. In the same line recent guidelines published by the European Society of Cardiology (ESC) recommend the usage of CCTA in rather patients at low intermediate (15–50%) pre-test probability for CAD, especially in candidates with low and stable heart rate and with therefore expected good image quality. In addition the presence of adequate technology and local expertise is important in this context (Montalescot et al., 2013).

**Table 4:** **Ability of representative 16-, 64- and >64-slice CCTA studies for the detection of anatomically significant CAD**.

**Study**	**Number of patients**	**Sensitivity (%)**	**Specificity (%)**	**PPV (%)**	**NPV (%)**	**Accuracy (%)**
**(A) 16-SLICE CCTA**						
Nieman et al., 2002	59	95	86	80	97	90
Ropers et al., 2003	77	92	93	79	97	93
Mollet et al., 2004	128	92	95	79	98	94
Kuettner et al., 2004	60	72	97	72	97	86
Dewey et al., 2006	129	82	90	90	95	87
Overview data	453	72–95	86–90	72–90	≥95	86–94
**(B) 64-SLICE CCTA**						
Meijboom et al., 2007	254	92	93	60	99	93
Shabestari et al., 2007	143	92	97	77	99	96
Leber et al., 2007	90	90	99	81	99	99
Ropers et al., 2007	100	90	98	79	99	98
Heuschmid et al., 2007	51	96	87	61	99	88
Miller et al., 2008	291	75	93	82	89	n.a
Overview data	929	75–96	87–99	60–82	89–99	88–99
**(C) >64-SLICE (128-, 256- & 320-SLICE) CCTA**						
Blank et al., 2014	65	95	54	47	96	n.a.
Petcherski et al., 2013	121	97	97	75	100	97
Chao et al., 2010	104	94	95	78	99	94
Korosoglou et al., 2010c	27	89	100	100	95	96
Dewey et al., 2009	30	78	98	75	99	97
Li et al., 2013	454	87	97	98	83	96
Achenbach et al., 2011	50	92	98	74	99	95
Overview data	851	78–97	54–100	47–100	≥95	≥94

More recent studies, performed with >64-slice CT scanners confirmed the high negative predictive value of CCTA for the exclusion of anatomically significant CAD (Dewey et al., 2009; Chao et al., 2010; Korosoglou et al., 2010c; Achenbach et al., 2011; Li et al., 2013; Petcherski et al., 2013; Blank et al., 2014). In this regard, the recent introduction of 640-slice CT scanners allows for the acquisition of the whole coronary artery tree within one heart beat so that no stitching is required during image reconstruction. The use of adaptive iterative dose reduction algorithms with this systems was shown to allow for coronary CTA with relatively low radiation exposure of 2.0–2.6 mSv (Yoo et al., 2013; Di Cesare et al., 2014).

**Figure 7:**
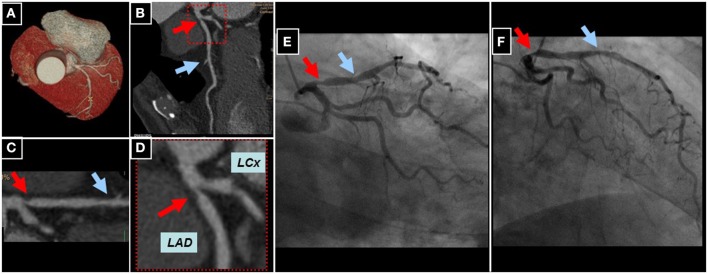
**Whole heart reconstruction **(A)** of a male patient undergoing CCTA (256-slice iCT scanner, Philips Medical Systems, Best, The Netherlands) for suspected CAD due to atypical angina**. A high-grade lesion of the proximal (red arrows) and an intermediate lesion (blue arrows) of the mid LAD can be appreciated in corresponding longitudinal **(B)** and curved multi-planar reconstructions **(C,D)**. Subsequent invasive coronary angiography confirmed the presence of CAD in the proximal and mid LAD **(E,F)**.

An example of a high-grade proximal LAD lesion using 256-slice CCTA can be appreciated in a male patient with atypical angina using whole-heart (Figure 7A), longitudinal (Figure 7B), and multi-planar reconstructions (Figures 7C,D). Subsequent invasive coronary angiography confirmed the presence of the proximal LAD lesion (Figures 7E,F).

Currently, an individual patient data meta-analysis is intended within the PROSPERO database, which will include individual patient data originating from studies comparing CCTA to invasive X-Ray angiography (Schuetz et al., 2013). This meta-analysis will possibly address the important question of which patients benefit most from CCTA in terms of clinical presentation and risk profile. To date, the most frequent indication for CCTA is found in patients with low or intermediate likelihood for CAD in order to avoid unnecessary invasive procedures. Thus, clinicians and radiologist should be aware of limiting factors such as severe coronary calcification (Gitsioudis et al., 2014), prior PCI and stent placement, arrhythmia and high body-mass-index (Carrabba et al., 2010), which may result in a higher number of false positive results, leading to unnecessary invasive diagnostic procedures.

The cost-effectiveness of CCTA for the diagnostic work-up of patients with suspected CAD was systematically evaluated in a recent meta-analysis (Zeb et al., 2014). Hereby, CCTA represented a cost-effective diagnostic strategy in the evaluation of patients with stable chest pain and CAD prevalence of 10–50%, as well as in patients with acute chest pain and low risk for obstructive CAD. In such patients CCTA may be associated with less downstream testing, expedited patient management and safe exclusion of acute coronary syndromes. In patients with CAD prevalence of ≥70% however, invasive angiography should rather represent the first line diagnostic modality. In the same line the ROMICAT trial previously demonstrated that in patients with symptoms suggestive of coronary syndromes, incorporating CCTA into a triage strategy improved the efficiency of clinical decision making, without resulting in a significant increase of the overall costs of care (Hoffmann et al., 2012).

### Assessment of coronary plaque composition and risk stratification

#### Assessment of plaque composition

**Figure 8:**
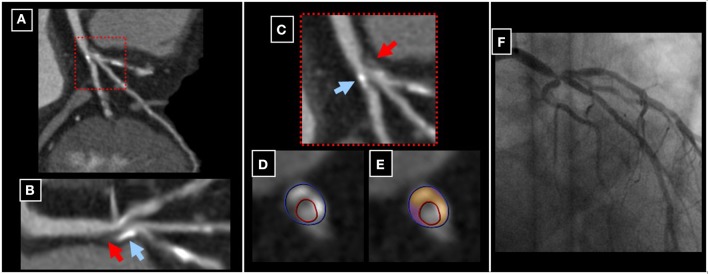
**A party calcified plaque in the left main coronary artery can be appreciated in corresponding multi-planar (A,C), longitudinal (B) and circumferential (D,E) CCTA reconstructions (red and blue arrows in B and C pointing to non-calcified and calcified components of the plaque, respectively)**. The presence of a high-grade left main lesion was subsequently confirmed by invasive coronary angiography **(F)**.

Despite the fact that conventional X-ray coronary angiography still remains the gold standard for detection of CAD, this technique provides limited information on the composition of atherosclerotic plaque (Libby, 2002). CCTA on the other hand, provides the non-invasive visualization of coronary plaque composition with high precision (Achenbach et al., 2004; Voros et al., 2011). First generation CCTA scanners offered limited ability for the reliable detection of coronary lesions due to lower spatial and temporal resolution. The development of multi-slice CT scanners with faster gantry rotation speed, Z-direction focal-spot sampling and spherical detector design however, helped to circumvent such limitations (Ong et al., 2006; Stolzmann et al., 2008; Voros, 2009; Chao et al., 2010; Hsiao et al., 2010; de Graaf et al., 2010). In addition, recent software developments with dedicated post-processing tools constituted major steps toward the reliable and quantitative assessment of atherosclerotic plaque composition (Mollet et al., 2005; Hamon et al., 2006; Ropers et al., 2006; Pohle et al., 2007; Schmid et al., 2008; Korosoglou et al., 2010c). Such software tools allow for the quantitative assessment of (Murray and Lopez, 1997) total plaque volume (Myerburg et al., 1997) distribution of calcified and non-calcified content within the plaque and (Naghavi et al., 2003) mean plaque density in Hounsfield Units (HU) with low inter-observer variability and within a reasonable time-spent (Korosoglou et al., 2010c; Rinehart et al., 2011). Current guidelines of the Society of Cardiac Computed Tomography (SCCT) introduced a scheme for the visual characterization of different plaque types for clinical reporting (Raff et al., 2009). In general, the percentage of calcium content is <20% in non-calcified plaque, between 20 and 80% in party calcified (mixed) plaque and >80% in calcified plaque. The reproducibility of this qualitative assessment (calcified, non-calcified, mixed plaques) was shown to be good in terms of both intra- and inter-observer agreement (Lehman et al., 2009; Rinehart et al., 2011). According to the tissue specific attenuation properties, 3 different plaque components can potentially be distinguished, including (Murray and Lopez, 1997) lipid-rich (14–70 HU), (Myerburg et al., 1997) fibrotic (71–150 HU) and (Naghavi et al., 2003) calcified components (>150–200 HU) (Pohle et al., 2007). However, there is still a lack of a uniform attenuation cut-off values defining these tissue qualities due to overlapping attenuation intervals, so that lipid and fibrotic plaque components are often summarized as “non-calcified.” Representative examples of a partly calcified atherosclerotic coronary plaque can be appreciated in Figure 8. Hereby, the complexity of this plaque in the left main coronary artery can be appreciated in corresponding multi-planar (Figures 8A,C), longitudinal (Figure 8B) and circumferential (Figures 8D,E) CCTA reconstructions. The presence of a high-grade left main lesion can be appreciated by invasive coronary angiography (Figure 8F).

#### Estimation of clinical outcomes by CCTA

Several studies evaluated the ability of atherosclerotic plaque composition assessment by CCTA to estimate cardiac outcomes in patients with suspected or known CAD and clinically stable chest pain syndrome (Pundziute et al., 2007; Gaemperli et al., 2008; Aldrovandi et al., 2009; Carrigan et al., 2009; Gopal et al., 2009; Hadamitzky et al., 2009; Rubinshtein et al., 2009; van Werkhoven et al., 2009a, reviewed in Bamberg et al., 2011) and summarized in Table 5). The recently published meta-analysis, which systematically reviewed the findings of these studies, included 7335 patients with stable CAD (Bamberg et al., 2011). Hereby, the presence and extent of plaque and coronary lesions by CCTA were independent predictors of future cardiovascular events. Based on 252 outcome events (6% all-cause, 6% cardiovascular mortality, 23% non-fatal MI, 4% unstable angina and 62% coronary revascularization), the estimated hazard ratio was 10.7, indicating a more than 10-fold higher risk among patients with vs. without obstructive CAD. In addition, subjects with any coronary plaque were at ~4.5-fold risk for future events, compared to patients without plaque.

**Table 5:** **Studies investigating the ability of CCTA for the assessment of clinical outcomes by evaluation of (A) coronary artery stenosis and (B) atherosclerotic plaque**.

**Study**	**Number of patients**	**Median follow-up time (years)**	**Event rate in all patients (%)**	**Event rate in CT positive patients (%)**	**Event rate in CT negative patients (%)**	**Hazard ratios**
**(A) PREDICTION OF OUTCOMES BY CORONARY ARTERY STENOSIS**						
Pundziute et al., 2007	100	2.2	26	58	8	28.0
Gaemperli et al., 2008	220	1.2	23	51	2	12.7
Carrigan et al., 2009	227	2.3	3.5	13	0.5	9.8
Gopal et al., 2009	493	3.3	1.2	6.5	0.1	16.6
Hadamitzky et al., 2009	1150	1.5	1.2	3.3	0.3	16.1
Aldrovandi et al., 2009	187	2.0	5.4	24.3	1.0	34.9
Rubinshtein et al., 2009	545	1.5	6.4	14.5	1.0	10.9
van Werkhoven et al., 2009b	432	1.8	2.9	6.5	2.5	3.6
van Werkhoven et al., 2009a	316	1.8	2.2	6.0	1.7	3.5
Andreini et al., 2012	1304	4.3	2.6	19.9	0	4.8
Motoyama et al., 2009	1059	2.6	0.8	22.3	0.5	22.8
Hadamitzky et al., 2011	2223	2.3	0.9	2.9	0.3	13.5
**(B) PREDICTION OF OUTCOMES BY CORONARY PLAQUE**						
Pundziute et al., 2007	100	2.2	24	30	0	n.a.
Gaemperli et al., 2008	220	1.2	23	28	0	n.a.
Ostrom et al., 2008	2538	6.5	0.5	0.72	0.26	n.a.
van Werkhoven et al., 2009b	432	1.8	1.9	4.9	1.4	n.a.
van Werkhoven et al., 2009a	316	1.8	1.3	3.5	0.64	n.a.

In the recently published CONFIRM registry both lumen narrowing and plaque burden, especially in proximal coronary segments were predictive of cardiovascular mortality in patients with suspected CAD (Hadamitzky et al., 2013). In this study, patients with proximal segments with stenosis >50% exhibited higher mortality rates (*HR* = 1.46 with a 95%CI of 1.15–1.87) compared to those without proximal coronary stenosis. In this regard, the number of proximal segments with stenosis >50% and the number of proximal segments with mixed or calcified plaque were identified as the best predictors of outcome surpassing the value provided by standard clinical variables, including the Framingham and the Morise score. In addition, the value of risk assessment in patients with CAD using a CCTA-based semi-automated plaque assessment has been recently shown (Versteylen et al., 2013). In this regard, quantitatively assessed total plaque and total non-calcified volume were predictive for future acute coronary syndromes, providing incremental value over clinical risk profiling and conventional CCTA readings.

#### The role of biomarkers in combination with imaging (bio-imaging)

Fewer studies have investigated the complementary value of cardiac biomarkers to CCTA imaging findings for the estimation of cardiac outcomes so far. Several biomarkers are used in clinical routine together with clinical assessment and 12-lead ECG for the triage of patients with acute coronary syndrome (ACS). In this regard, cardiac troponins were shown to aid the diagnostic classification and risk stratification of such patients (Katus et al., 1991; Korosoglou et al., 2004; Thygesen et al., 2012). Recently, we and others demonstrated an association between atherosclerotic plaque composition by CCTA and high-sensitivity cardiac troponin even in non-ACS patients (Laufer et al., 2010; Korosoglou et al., 2011b). This association may be explained by chronic clinically silent rupture of non-calcified plaque, which results to embolization of atherosclerotic debris in the microcirculation and to consecutive myocardial micro-necrosis. In a further experimental setting, HMBG1 (high mobility group box 1) protein was found to be a critical mediator of acute ischemic injury, predicting adverse outcomes after myocardial infarction (Andrassy et al., 2008, 2011). In addition, we could show that HMBG1 serum levels are associated with coronary calcification and with non-calcified plaque composition in patients with suspected or known stable CAD (Andrassy et al., 2012).

C-reactive protein (CRP) on the other hand, was previously proposed as a central mediator in atherosclerotic plaque development and vascular inflammation (Zhang et al., 1999). However, further basic science research questioned such initially proposed direct atherogenic mechanisms (Clapp et al., 2005; Koike et al., 2009). In this regard, we and others demonstrated that serum levels of high sensitivity CRP are only weakly and not independently associated with plaque composition (Hamirani et al., 2008; Blaha et al., 2011; Korosoglou et al., 2011b). More specific inflammatory markers of such as HMBG1 could provide a stronger association with plaque formation in this context. In this regard, the recently published dal-PLAQUE study demonstrated that myeloperoxidase (MPO) levels are associated with carotid plaque inflammation, assessed using 18F-fluorodeoxyglucose positron emission tomography/computed tomography (FDG-PET/CT) (Duivenvoorden et al., 2013). An overview of studies within the bio-imaging area (Laufer et al., 2010; Blaha et al., 2011; Korosoglou et al., 2011b; Andrassy et al., 2012; Duivenvoorden et al., 2013; Nakazato et al., 2013; Voros et al., 2013) is presented in Table 6. Future studies are now warranted in order to investigate the ability of CCTA in combination with cardiac or inflammatory biomarkers for the individualized risk stratification of patients with presumably stable CAD.

**Table 6:** **“Bio-imaging” studies using coronary computed tomography and biomarkers in stable CAD patients**.

	**Bio-markers**	**CT Imaging technique**	**Number of patients**	**Main result/ conclusion**
Laufer et al., 2010	hsTnT	64-slice CT	615	HsTnT strongly is associated with CAD in patients without ACS.
Korosoglou et al., 2011b	hsTnT	256-slice CT	124	HsTnT is closely related to coronary plaque composition.
Blaha et al., 2011	hsCRP	4-slice CT for CAC	6762	hsCRP is not associated with coronary calcification.
Duivenvoorden et al., 2013	Myeloperoxidase (MPO), hsCRP	^18^FDG-PET/CT	130	MPO levels are associated with carotid plaque inflammation.
Andrassy et al., 2012	HMBG-1	256-slice CT	152	HMBG1 is associated with the atherosclerotic plaque composition.
Nakazato et al., 2013	LDL, HDL and total cholesterol	≥64-slice CT	4575	Non-HDL is associated with non-calcified coronary plaque.
Voros et al., 2013	ApoB, HDL, LDL	64-sl. MDCT IVUS/VH	60	ApoB and small HDL particles are associated with larger plaque burden and non-calcified plaque.

CAD, Coronary artery disease; LDL, low-density lipoprotein; HDL, high-density lipoprotein; ApoB, Apolipoprotein B; hsTnT, high-sensitive Troponin T; hsCRP, high-sensitive C-reactive protein; MPO, myeloperoxidase; CT, computer tomography; CAC, coronary artery calcification.

### Radiation exposure with CCTA

**Figure 9:**
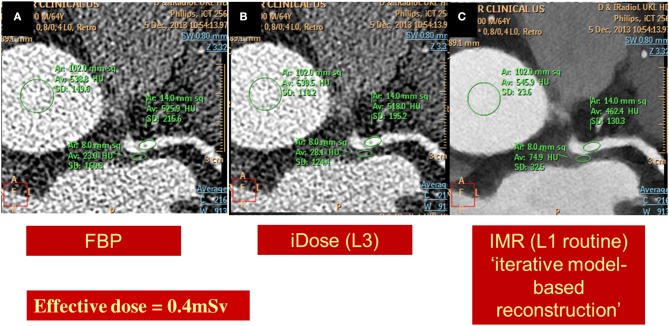
**Images of a left coronary artery, reconstructed using standard back filtered (A) and iterative algorithms [iDose in B and knowledge based iterative model reconstruction (IMR) in C] with a resultant radiation exposure of 0.4 mSv**. Diagnostic image quality and significant noise reduction is provided using IMR.

Optimal diagnostic image quality with a minimum dosage of radiation exposure for the patients still represents a major challenge. In this regard, radiation exposure with CCTA still raises concerns among physicians, as it may be associated with non-negligible lifetime attributable risk of breast or lung cancer, particularly in women and in younger patients (Einstein et al., 2007). In one of the first studies, which systematically investigated the radiation dose of CCTA in routine clinical practice, median CCTA doses were shown to be extremely different among different study sites and CT scanners (Hausleiter et al., 2009). Since then several strategies have been developed to reduce radiation dose, including dose modulation techniques, prospective ECG triggering, low-tube voltage CT imaging and iterative reconstruction algorithms (Hausleiter et al., 2010; Hosch et al., 2011, 2012, 2013). In this regard, the PROTECTION I study demonstrated that prospective ECG-triggered CCTA can significantly reduce radiation dose by ~70%, without influencing image quality when compared to standard retrospective scans in patients with a low and stable heart rate (Bischoff et al., 2010). These results were subsequently confirmed in the multicenter, multivendor, randomized PROTECTION III study (Hausleiter et al., 2012). The PROTECTION II study on the other hand, demonstrated that 100kV low tube voltage CCTA is associated with a further significant ~30% reduction of the resultant radiation exposure with simultaneously maintained diagnostic image quality (Hausleiter et al., 2010). More recently, the introduction of prospectively triggered high-pitch spiral CCTA allowed the acquisition of diagnostic images with a resultant radiation exposure of <1mSv in non-obese patients with low and stable heart rates (Achenbach et al., 2011). The combination of this new high-pitch spiral technology with low tube voltage and iterative reconstruction can further dramatically reduce radiation exposure with CCTA, achieving an effective dose below 0.1mSv with sufficient image quality (Schuhbaeck et al., 2013). However, such an ultra-low protocol may be associated with a non-negligible number of non-diagnostic segments even in non-obese patients with stable heart rates. In general CCTA can be nowadays assessed with a radiation exposure of ~0.5–1 mSv in most patients, by combining the use of prospective ECG-triggering, low-tube voltage, BMI-adapted protocols and iterative reconstruction algorithms. Representative images of a left coronary artery, reconstructed using standard back filtered (Figure 9A) and iterative algorithms (Figures 9B,C) with a resultant radiation exposure of 0.4 mSv can be appreciated in Figure 9.

In addition, the value of calcium scoring scans as a filter prior to CCTA, in order to identify patients with severe calcification was recently shown to be limited in younger patients with intermediate risk profile. Omitting such calcium scoring pre-scans in younger patients can contribute to further absolute dose reduction of ~0.75–1.0 mSv with cardiac CT studies (Gitsioudis et al., 2014). This is important in light of the clinical applicability of current low-dose CCTA protocols (as shown in Figure 9) and of the fact that additional calcium scoring scans resulted in additional DNA double-strand breaks in recent clinical trials (Brand et al., 2012).

## Summary and conclusions

Coronary computed tomography angiography and CMR can both be regarded as in the meanwhile clinically well-established techniques for the diagnostic classification and risk stratification of patients with suspected or known CAD. The strengths of CCTA are its ability (1) to non-invasively visualize moving coronary vessels with high spatial resolution, excluding significant CAD with high precision and (2) to assess the composition of atherosclerotic plaque components. Its main limitation is the resultant radiation exposure for the patients, which limits its serial applicability particularly in younger patients. However, scientist, clinicians, and manufacturers managed significant reductions of radiation exposure in this field within the last 5 years. The strength of CMR on the other hand, is its ability to assess inducible wall motion abnormalities and perfusion defects during stress testing aiding the detection of functionally significant CAD. Thus, it represents the optimal technique for the risk stratification of patients with suspected CAD and for guiding revascularization procedures in patients with diagnosed CAD by CCTA or invasive angiography. Current guidelines encourage the liberal use of both CCTA and CMR as first choice modalities for the diagnostic work-up of patients with low and intermediate likelihood for CAD in experienced centers.

### Conflict of interest statement

The authors declare that the research was conducted in the absence of any commercial or financial relationships that could be construed as a potential conflict of interest.
